# Comparison of adherence between fixed and unfixed topical combination glaucoma therapies using Japanese healthcare/pharmacy claims database: a retrospective non-interventional cohort study

**DOI:** 10.1186/s12886-021-01813-w

**Published:** 2021-01-21

**Authors:** Chikako Shirai, Nobushige Matsuoka, Toru Nakazawa

**Affiliations:** 1 Medical Affairs, Pfizer Japan Inc., 3-22-7 Yoyogi, Shibuya-ku, Tokyo, 151-8589 Japan; 2 Biometrics & Data Management, Pfizer R&D Japan G.K, 3-22-7 Yoyogi, Shibuya-ku, Tokyo, 151-8589 Japan; 3 Department of Ophthalmology, Tohoku University Graduate School of Medicine, 1-1 Seiryo-machi, Aoba-ku, Sendai, Miyagi 980-8574 Japan

**Keywords:** Adherence, Persistence, Glaucoma, Fixed-combination eye drops, Claims database, Latanoprost

## Abstract

**Background:**

Adherence to chronic therapies is crucial to prevent the progression of disease, such as glaucoma. However, only a limited number of studies have investigated them using real-world data in Japan. This study aimed to evaluate Japanese patients’ adherence to fixed- and unfixed-combination eye drops as a second-line therapy for glaucoma in real-world practice.

**Methods:**

This retrospective, non-interventional cohort study utilized a commercially available Japanese healthcare database (MinaCare database). Medical/pharmacy claims data were collected from 2011 to 2016. The primary endpoint was adherence to medications, assessed by proportion of days covered (PDC) with medication during a 12-month post-index period. Meanwhile, the secondary endpoints included the persistence rate.

**Results:**

A total of 738 patients were included in this study: 309 and 329 in the fixed- and unfixed-combination cohorts, respectively. Prostaglandin analog (PG)/β-blocker (BB) was most commonly claimed in 241/309 (78.0%) patients in the fixed-combination cohort. In the unfixed-combination cohort, PG and BB were claimed in 130/329 (39.5%) patients, whereas PG and α2-agonist were claimed in 87/329 (26.4%) patients. Patients were more adherent to the fixed-combination than the unfixed-combinations (mean PDCs [SD], 79.1% [32.1] vs. 62.2% [38.0]; *P* < 0.0001). The proportion of patients with good adherence (PDC ≥ 80%) was also higher in the fixed-combination cohort (69.6%) than in the unfixed-combination cohort (48.6%) (*P* < 0.0001). During the 12-month post-index period, the persistence rate was higher in the fixed-combination cohort than in the unfixed-combination cohort (47.6% [95% confidence intervals (CI): 41.9–53.0] vs. 24.9% [95% CI: 20.4–29.7], *P* < 0.0001).

**Conclusions:**

Japanese patients with glaucoma preferred the fixed-combination therapies over the unfixed-combination therapies. Hence, fixed-combination therapies would contribute to the improvement of adherence.

## Background

Glaucoma is the most common causative disease of blindness in Japan [1]. One of the critical risk factors for glaucoma is the intraocular pressure (IOP) [2–5]. IOP-lowering eye drops and surgeries, such as selective laser trabeculoplasty (SLT) and minimally invasive glaucoma surgery (MIGS), have been developed and used for treatment. Consequently, glaucoma has become manageable, provided that it is diagnosed early and the treatment is implemented in a timely manner to prevent the development and delay the progression of the disease [6, 7]. Recently a randomized study, called the LiGHT study, compared the clinical effectiveness as well as cost-effectiveness between the eye drops and the SLT groups and demonstrated that SLT is cost effective, suggesting that SLT should be offered as a first-line treatment for open-angle glaucoma and ocular hypertension [8]. However, IOP-lowering eye drops still remain a first-line treatment option in many countries including Japan.

The treatment for glaucoma is generally started with a monotherapy, such as prostaglandin analogs (PG) or β-blockers (BB). If the first monotherapy is ineffective or intolerable, switching to another monotherapy is preferred. Whenever a monotherapy does not reach the target IOP or the target must be lowered as the disease progresses, a concomitant therapy with a medication that has a different mechanism of action should be considered. If such treatment regimens are successful, patients can avoid laser intervention and surgery. However, adherence to the therapy should be crucially improved for a successful glaucoma treatment [9], considering that nonadherence to glaucoma therapies has been a serious concern [10, 11].

Risk factors for nonadherence to glaucoma therapies include patients’ problems (financial concern, poor understanding of the disease, and frequent eye drop application), healthcare providers’ issues (insufficient instruction/explanation and assessment of each patient’s conditions), and the relationship between patients and physicians (lack of communication and trust) [12–14]. A previous study reported that a fixed-combination therapy improved the long-term adherence of patients with glaucoma compared with the unfixed therapy [15]. According to a nationwide survey performed in 2011–2012 in Japan using a questionnaire, good adherence to IOP-lowering eye drops was observed in 72.4% of patients [16]. However, these risk factors and adherence rates were based on the data obtained from clinical studies under controlled conditions or an experimental setting. Therefore, patients’ adherence to the therapies should be investigated, and the risk factors in ordinary clinical settings should be determined using real-world data.

In several previous studies, adherence to glaucoma therapies was evaluated using pharmacy claims data [17–22]. Unfortunately, to our knowledge, no such study has been conducted in Japan. Therefore, this study aimed to compare patients’ adherence between a fixed-combination therapy and an unfixed-combination therapy and to identify risk factors for nonadherence in Japanese patients with glaucoma by using a healthcare database.

## Methods

### Study design

This retrospective cohort study utilized a commercially available Japanese administrative healthcare database (MinaCare Co., Ltd., Tokyo, Japan) to investigate the adherence of patients to fixed-combination and unfixed-combination therapies for glaucoma treatment after switching from a monotherapy. The study consisted of a 12-month pre-index period, an index date (defined as a first prescription day of either when a single eye drop was first switched to a fixed-combination therapy [fixed-combination cohort] or when the second eye drop was added to a single eye drop initially [unfixed-combination cohort]), and a 12-month post-index period. Patients without claims for 12 months after the index date and those who claimed the third drug in addition to a fixed-combination therapy or in addition to an unfixed-combination therapy indicated withdrawal. Approval for this research by an ethical review committee and informed consent of each subject were not required because studies using only unlinkably anonymized data are outside the scope of “Ethical Guidelines for Medical and Health Research Involving Human Subjects” [23] set by the Japanese government.

### Data source and study cohort

The MinaCare database includes anonymized data on both health checkup and medical/pharmacy claims of workers and their family members in a wide range of age groups below the age of 75 years [24, 25]. Maintained by MinaCare Co., Ltd., this database is updated periodically with newly available data obtained from the employment-based health insurance groups. It includes the data of 6.3 million uniquely identified individuals as of April 2017 and has recorded the information of 1.8 million patients from April 2016 to March 2017, covering 1.7% of the Japanese population under the age of 75 years [26]. Furthermore, it is generally consistent with two national databases and is useful as it has low selection bias and large sample size with wide age distribution; however, it only targets national-wide big corporations, such as manufacturing, food, information transportation, and energy industries, and does not include individuals in the primary industry (agriculture, fisheries, forestry, etc.) or those who are self-employed [24].

Patients were extracted from the database with the ICD10 diagnosis codes H401 (normal tension glaucoma, primary open-angle glaucoma, and open-angle glaucoma) and H409 (unspecified glaucoma) and with prescription claims for the eye drops to treat glaucoma between April 1, 2011 and March 31, 2016. In addition, we extracted the clinical laboratory data, such as anthropometric measurements, blood pressure, blood glucose levels, blood lipid levels, liver function test values, hematologic values, and urine test values, from the health checkup database. We included those who had received monotherapy for 1 year or longer without surgery including laser treatment and then switched to a fixed-combination therapy as second-line treatment (the fixed-combination cohort) or those who received an additional eye drop, such as timolol as an addition to latanoprost or vice versa, as second-line treatment (the unfixed-combination cohort). The first prescription date of the second-line therapy was defined as the index date. Patients without prescription of a fixed or unfixed-combination therapy, with history of surgery or laser surgery for glaucoma, or with pre-index monotherapy for < 12 months were excluded.

### Study endpoints

The primary endpoint was medication adherence, which was assessed by the proportion of days covered (PDC) with medication over the 12-month post-index period. The a priori definition of adherence cutpoint was 80%. Meanwhile, the secondary endpoints were related to medication persistence, defined as the act of continuing the use of claimed eye drops, and included persistence rate and its duration, a distribution of patients according to the type of claimed eye drops at the index date and switching patterns of eye drops during the 12-month post-index period. In addition, the risk factors for PDC < 80% were analyzed.

### Assessments

Subject background characteristics included sex, age, body weight, height, body mass index, smoking status/history, health examination results, comorbidity, residence region, the number of eye drop bottles consumed, and eye drop types. These characteristics were summarized in all patients and in each cohort. Moreover, residence regions were classified into eight regions from Hokkaido to Kyushu including Okinawa [27], two areas (East Japan vs. West Japan) [28], and city sizes (big cities including Tokyo and 20 ordinance-designated cities vs. the others) [29]. We also identified the PDC over 12 months after the index date in all patients and each cohort, the risk factors for PDC < 80%, and the persistence rate.

To address the possible variations in the dosage and prescribed period reported on a claim for glaucoma medication, we made adjustments based on the unit volume of the formulation. For example, if the dosage was “7.5” and the period was “1” in a claim, we converted them to “2.5 mL (approved bottle) × 3 bottles” prescribed for 3 months because one bottle (2.5 mL for once-daily formulations or 5 mL twice-or-more-daily formulations) is enough to apply one eye for more than 30 days but is correspondent to 30 days dispensed. The general recommendation of the use-by period of opened eye drop bottles is generally 4 weeks in Japan. Accordingly, the patients were permitted a 30-day grace period to obtain the next prescription. In addition, if one prescription consists of ≥7 bottles, the patient was considered receiving treatment of both eyes. Therefore, a PDC value was calculated using the following formula:

$$ \mathrm{PDC}\ \left(\%\right)=\left(\mathrm{total}\ \mathrm{prescription}\ \mathrm{days}\ \mathrm{during}\ \mathrm{the}\ 365-\mathrm{day}\ \mathrm{assessment}/365\ \mathrm{days}\right)\times 100 $$

In this study, the PDC is calculated according to the period only prescribed with two-drug fixed-combination drops or two-drug unfixed-combination therapies, regardless of switching drug class [e.g., PG + BB to PG+ carbonic anhydrase inhibitor (CAI)].

Persistence refers to the act of continuing index therapy (i.e., a fixed-combination therapy or an unfixed-combination therapy). The definition of “persistence” is the duration in days from the index date (the first prescription date of the index therapy) to the last prescription date + prescription days or to the discontinuation date allowing a 30-day grace period. In the case of one drug addition to the combination of two drugs (e.g., PG + BB to PG + BB + AA), it was considered as “discontinued/withdrawn.” Likewise, in a subset analysis using specific drug classes, no prescription record for more than 30 days or switched or changed prescriptions were treated as “discontinued.” The cumulative discontinuation rate was analyzed based on the time to discontinuation and was estimated using the Kaplan–Meier method. Patients who continued the treatment for 12 months were treated as censored at 12 months. The persistence rate at the evaluated time point was calculated as follows:

$$ \mathrm{Persistence}\ \mathrm{rate}\ \left(\%\right)=100\%-\mathrm{cumulative}\ \mathrm{discontinuation}\ \mathrm{rate} $$

### Statistical analysis

We presented summary statistics (mean and standard deviation [SD] or a number and a proportion of patients) for the patients’ background characteristics and PDC. Using *t*-test, we compared the mean PDC values between two cohorts. Furthermore, proportions of patients with or without ≥80% PDC between two cohorts were compared using Pearson’s χ^2^ tests. Bonferroni correction was used to adjust for multiplicity (m = 9, where m is the total number of statistical tests in this study). A *p*-value = < 0.00556 (0.05 / 9) was considered statistically significant for comparisons between the groups. We also explored the risk factors for nonadherence to the medications (PDC < 80%) by using a univariate logistic regression model. Subsequently, multivariate analyses were performed for those with *P* < 0.1, followed by a stepwise (forward and backward) logistic regression model. The odds ratios (OR) and Wald 95% confidence intervals (CIs) were presented. The subject persistence rates at 6 and 12 months with a pointwise 95% CI were estimated using the Kaplan–Meier method, and the differences between the two treatment cohorts were tested by log-rank test. All the statistical analyses were performed using the SAS version 9.4 (SAS Institute Inc., Cary, NC, USA).

## Results

**Fig. 1:**
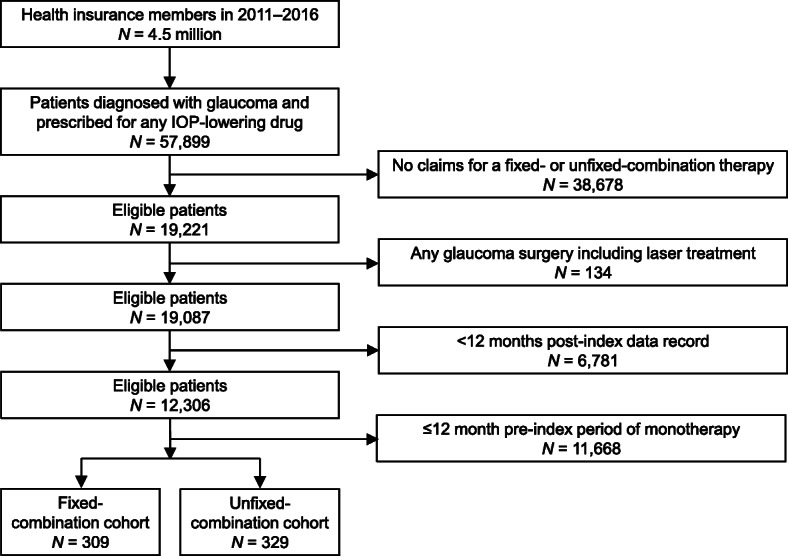
Flowchart for data extraction. IOP, intraocular pressure

In total, 57,899 patients were diagnosed with glaucoma and prescribed with any IOP-lowering drug in the database, of whom 309 patients were in the fixed-combination cohort and 329 patients were in the unfixed-combination cohort (Fig. 1). The two cohorts had similar baseline demographic and clinical characteristics (Table 1). The mean age was 56–57 years and clinical measurement values were within the normal ranges. Comorbidities reported in > 20% of patients were hypertension, hyperlipidemia, diabetes mellitus, and cancer. In both cohorts, over 80% of patients lived in East Japan (especially residents of Tokyo or large cities).

**Table 1:** Baseline demographic and clinical characteristics of subjects

*Variable*	*Fixed combination (N = 309)*		*Unfixed combination (N = 329)*	
	*n*	*Percentage or mean ± SD*	*n*	*Percentage or mean ± SD*
Sex, male	170	55.0	188	57.1
Age (years)	309	56.5 ± 10.2	329	57.1 ± 10.2
Body weight (kg)	210	61.8 ± 12.5	219	61.6 ± 11.0
Height (cm)	218	163.9 ± 9.2	226	164.4 ± 8.9
Body mass index (kg/m^2^)	210	22.9 ± 3.5	219	22.7 ± 3.1
Smoking				
Yes	33	10.7	32	9.7
No	178	57.6	193	58.7
Medication for other indications				
Yes	63	20.4	74	22.5
No	146	47.2	149	45.3
Fasting blood glucose (mg/dL)	200	97.0 ± 17.4	213	97.8 ± 15.4
HbA1c (NGSP) (%)	205	5.6 ± 0.6	216	5.6 ± 0.5
Urine glucose				
Negative (−)	208	67.3	221	67.2
Positive (≥±)	4	1.3	2	0.6
Urine protein				
Negative (−)	192	62.1	207	62.9
Positive (≥±)	24	7.8	19	5.8
Antihypertensive drug				
Yes	46	14.9	52	15.8
No	163	52.8	171	52.0
Systolic blood pressure (mm Hg)	218	119.9 ± 15.5	226	120.3 ± 16.2
Diastolic blood pressure (mm Hg)	218	74.5 ± 11.3	226	75.6 ± 11.2
Total cholesterol (mg/dL)	64	207.3 ± 32.0	53	201.7 ± 35.9
Triglycerides (mg/dL)	217	107.9 ± 64.8	226	101.3 ± 66.6
HDL cholesterol (mg/dL)	217	64.6 ± 16.7	226	65.0 ± 16.9
LDL cholesterol (mg/dL)	217	124.4 ± 26.9	226	126.2 ± 31.2
Aspartate transaminase (U/L)	217	23.7 ± 9.3	226	23.0 ± 8.8
Alanine aminotransferase (U/L)	217	23.3 ± 14.6	226	23.5 ± 13.6
γ-GTP (U/L)	216	41.4 ± 49.1	226	37.6 ± 41.6
Common comorbidities				
Hypertension	92	29.8	110	33.4
Cancer	90	29.1	114	34.7
Hyperlipidemia	87	28.2	111	33.7
Diabetes mellitus	85	27.5	97	29.5
Insomnia	44	14.2	44	13.4
Liver disorder	43	13.9	44	13.4
Asthma or COPD	41	13.3	66	20.1
Gastrointestinal ulcer	38	12.3	41	12.5
Atherosclerosis or PAOD	31	10.0	38	11.6
Anemia	30	9.7	33	10.0
Coronary artery disease	26	8.4	33	10.0
Heart failure	22	7.1	35	10.6
Locations				
8 regions				
Hokkaido	7	2.3	3	0.9
Tohoku	14	4.5	6	1.8
Kanto	176	57.0	207	62.9
Chubu	38	12.3	37	11.2
Kinki	38	12.3	36	10.9
Chugoku	10	3.2	9	2.7
Shikoku	2	0.6	2	0.6
Kyushu	24	7.8	29	8.8
Areas				
East Japan	210	68.0	220	66.9
West Japan	99	32.0	109	33.1
City size				
Tokyo and all GODMCs	255	82.5	289	87.8
Other than GODMCs	54	17.5	40	12.2

*HBA1c* hemoglobin A1c, *NGSP* National Glycohemoglobin Standardization Program, *HDL* high-density lipoprotein, *LDL* low-density lipoprotein, *COPD* chronic obstructive pulmonary disease, *γ-GTP* γ-glutamyl transpeptidase, *PAOD* peripheral arterial occlusive disease, *GODMCs* government ordinance-designed major cities

**Fig. 2:**
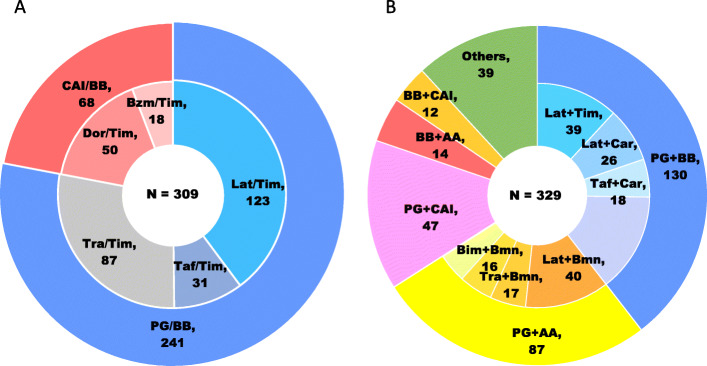
Drug class distribution of prescribed eye drops on the index date in the fixed-combination (**a**) and unfixed-combination (**b**) cohorts. AA, α2-agonist; BB, β-blocker; Bim, bimatoprost; Bmn, brimonidine; Bzm, brinzolamide; CAI, carbonic anhydrase inhibitor; Car, carteolol; Dor, dorzolamide; Lat, latanoprost; PG, prostaglandin analog, Taf, tafluprost; Tim, timolol; Tra, travoprost

The eye drops claimed at the index date are summarized in Fig. 2. In the fixed-combination cohort, PG/BB (78.0%) and CAI/BB (22.0%) were used by drug class, and the most commonly used eye drop was latanoprost/timolol (39.8%) by generic name. In the unfixed-combination cohort, the top three most frequently administered drug combinations were PG + BB (39.5%), PG + α2-agonist (AA) (26.4%), and PG + CAI (14.3%) by drug class; the most common drugs were latanoprost and brimonidine (12.2%), followed by latanoprost and timolol (11.9%) by generic name.

**Fig. 3:**
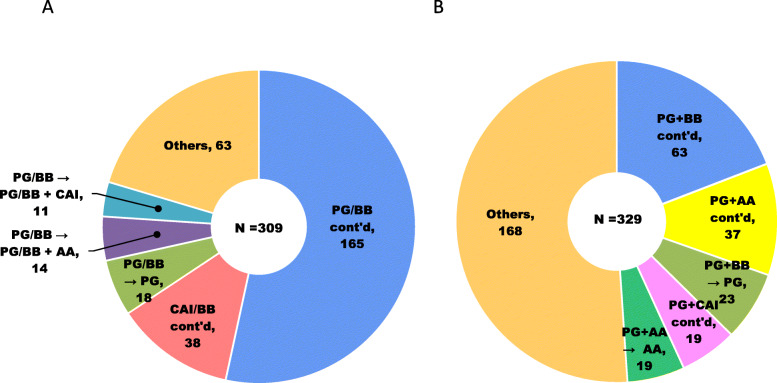
Switching patterns from the fixed-combination (**a**) and unfixed-combination (**b**) eye drops during the 12-month post-index period. AA, α2-agonist; BB, β-blocker; CAI, carbonic anhydrase inhibitor; cont’d, continued; PG, prostaglandin analog

Common patterns of switching eye drops during the post-index period are shown in Fig. 3. In the fixed-combination cohort, out of 241 patients prescribed with PG/BB, 165 (68.5%) continued the same drug class, and out of 68 patients prescribed with CAI/BB, 38 (55.9%) continued the same drug class. Other major switching patterns were the addition of AA or CAI. In the unfixed cohort, the same drug class combination therapies were continued by only less than 50% of patients, regardless of the kind of combination.

The mean PDC over the 12-month post-index period was 70.4% in all the patients, 79.1% in the fixed-combination cohort, and 62.2% in the unfixed-combination cohort; hence, the PDC in the fixed-combination cohort was significantly higher than that in the unfixed-combination cohort (*P* < 0.0001) (Table 2). High adherence (PDC ≥ 80%) was observed in 58.8% of all the patients, 69.6% in the fixed-combination cohort, and 48.6% in the unfixed-combination cohort; hence, it was significantly higher in the fixed-combination cohort than in the unfixed-combination cohort (*P* < 0.0001). As for the popular combination of latanoprost and timolol, the mean PDC was 82.9% in the fixed-combination cohort and 66.5% in the unfixed-combination cohort; hence, it was higher in the fixed-combination cohort (*P* = 0.0191) (Table 3), but it was not significant (NS). A proportion of patients with PDC ≥ 80% was 75.6% in the fixed-combination cohort and 53.8% in the unfixed-combination cohort, showing that it was higher in the fixed-combination cohort (*P* = 0.0095, NS). Furthermore, when compared between the PG/BB and CAI/BB of the same fixed-combination cohort, PG/BB had an approximately 10% higher mean PDC value (*P* = 0.0438, NS) and an approximately 15% higher proportion of patients with good adherence (*P* = 0.0131, NS) (Table 4).

**Table 2:** Adherence to fixed- and unfixed-combination therapies during the 12-month post-index period

	*All patients* *N = 638*	*Fixed combination* *N = 309*	*Unfixed combination* *N = 329*	
PDC, mean ± SD (%)	70.4 ± 362	79.1 ± 32.1	62.2 ± 38.0	P < 0.0001^a^
	n (%)	n (%)	n (%)	
80 + % PDC	375 (58.8)	215 (69.6)	160 (48.6)	P < 0.0001^b^
< 80% PDC	263 (41.2)	94 (30.4)	169 (51.4)	
60–79% PDC	46 (7.2)	16 (5.2)	30 (9.1)	
40–59% PDC	43 (6.7)	24 (7.8)	19 (5.8)	
20–39% PDC	54 (8.5)	19 (6.1)	35 (10.6)	
< 20% PDC	120 (18.8)	35 (11.3)	85 (25.8)	

*PDC* proportion of days covered

^a^, *t*-test (fixed vs. unfixed); ^b^, χ^2^ test (fixed vs. unfixed). Note: a *p*-value < 0.05 / 9 (= 0.00556) was considered statistically significant (by Bonferroni correction)

**Table 3:** Adherence to fixed- and unfixed-combination therapies with latanoprost and timolol during the 12-month post-index period

	*All patients* *N = 162*	*Fixed combination* *N = 123*	*Unfixed combination* *N = 39*	
PDC, mean ± SD (%)	78.9 ± 33.7	82.9 ± 31.1	66.5 ± 38.6	P = 0.0191^a^
	n (%)	n (%)	n (%)	
80 + % PDC	114 (70.4)	93 (75.6)	21 (53.8)	P = 0.0095^b^
< 80% PDC	48 (29.6)	30 (24.4)	18 (46.2)	
60–79% PDC	8 (4.9)	6 (4.9)	2 (5.1)	
40–59% PDC	9 (5.6)	5 (4.1)	4 (10.3)	
20–39% PDC	10 (6.2)	7 (5.7)	3 (7.7)	
< 20% PDC	21 (13.0)	12 (9.8)	9 (23.1)	

*PDC* proportion of days covered

^a^, *t*-test (fixed vs. unfixed); ^b^, χ^2^ test (fixed vs. unfixed). Note: a p-value < 0.05 / 9 (= 0.00556) was considered statistically significant (by Bonferroni correction)

**Table 4:** Comparison of adherences to fixed-combination therapies during the 12-month post-index period

	*PG/BB* *N = 241*	*CAI/BB* *N = 68*	
PDC, mean ± SD (%)	81.2 ± 31.0	71.6 ± 34.9	P = 0.0438^a^
	n (%)	n (%)	
80 + % PDC	176 (73.0)	39 (57.4)	P = 0.0131^b^
< 80% PDC	65 (27.0)	29 (42.6)	
60–79% PDC	12 (5.0)	4 (5.9)	
40–59% PDC	15 (6.2)	9 (13.2)	
20–39% PDC	13 (5.4)	6 (8.8)	
< 20% PDC	25 (10.4)	10 (14.7)	

*PDC* proportion of days covered, *PG* prostaglandin analogs, *BB* β-blockers, *CAI* carbonic anhydrase inhibitors

^a^, *t*-test (PG/BB vs. CAI/BB); ^b^, χ^2^ test (PG/BB vs. CAI/BB). Note: a *p*-value < 0.05 / 9 (= 0.00556) was considered statistically significant (by Bonferroni correction)

Univariate analyses identified treatment (fixed-combination vs. unfixed-combination) and hemoglobin A1c (HbA1c) as potential risk factors for nonadherence (*P* < 0.1) (Table 5). Conversely, variables, such as age, sex, body mass index, smoking habit, hospital locations, and comorbidities, were not associated with nonadherence. In multivariate analyses, treatment (adjusted OR: 0.388 [95% CI: 0.259, 0.583], *P* < 0.0001) and HbA1c (adjusted OR: 1.606 per 1% [95% CI: 1.105, 2.333], *P* = 0.0130) were also considered as significant risk factors.

**Table 5:** Risk factors for nonadherence (PDC < 80%) to medications

Variable	Univariate	Multivariate	Odds ratio (95% CI)
	*P* Value	P Value	
Fixed vs. unfixed combination therapies	< 0.0001	< 0.0001	0.388 (0.259, 0.583)
Sex	0.4583		
Age	0.6787		
Body weight	0.9582		
Height	0.4747		
Body mass index	0.5763		
Smoking habit	0.5399		
Medication for other disease	0.9166		
Antihypertensive drug	0.6910		
Fasting blood glucose	0.4806		
HbA1c	0.0219	0.0130	1.606 (1.105, 2.333)
Urine glucose	0.2091		
Urine protein	0.3810		
Systolic blood pressure	0.3303		
Diastolic blood pressure	0.6046		
Total cholesterol	0.3382		
Triglyceride	0.7453		
47 prefectures	> 0.9999		
8 regions	0.7097		
East vs. West Japan	0.1569		
City size	0.4610		
Hypertension	0.5190		
Cancer	0.3087		
Hyperlipidemia	0.8102		
Diabetes mellitus	0.4790		
Insomnia	0.1181		
Liver disorder	0.9745		
Asthma or COPD	0.6839		
Gastrointestinal ulcer	0.7023		
Atherosclerosis or PAOD	0.8851		
Anemia	0.1098		
Coronary artery disease	0.4576		
Heart failure	0.1229		

*CI* confidence interval, *COPD* chronic obstructive pulmonary disease, *PAOD* peripheral arterial occlusive disease, *PDC* proportion of days covered

**Fig. 4:**
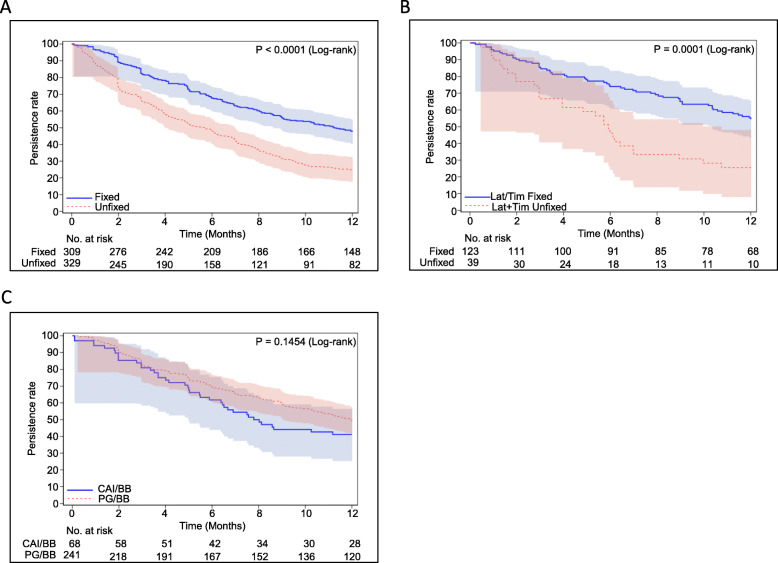
Kaplan–Meier survival curves for the treatment persistence with fixed- vs. unfixed-combination therapies (**a**), fixed- vs. unfixed-combination therapies with Lat and Tim (**b**), and fixed-combination therapies with PG and BB vs. CAI and BB (**c**). Bars were Hall-Wellner bands. BB, β-blocker; CAI, carbonic anhydrase inhibitor; Lat, latanoprost; PG, prostaglandin analog; Tim, timolol

Kaplan–Meier curves of persistence are presented in Fig. 4a. According to nonoverlapping 95% CIs, more patients in the fixed-combination cohort than in the unfixed cohort continued their treatment after 2 months post-index, with a significant difference between the two cohorts (log-rank test, *P* < 0.0001). The persistence rates at 6 and 12 months post-index were approximately 67.6% (95% CI: 62.1, 72.5) and 47.6% (95% CI: 41.9, 53.0), respectively, in the fixed-combination cohort, and 48.0% (95% CI: 42.5, 53.3) and 24.9% (95% CI: 20.4, 29.7), respectively, in the unfixed-combination cohort.

Likewise, the persistence rate of the fixed-combination therapy with latanoprost and timolol was significantly higher than that of the unfixed-combination cohort (log-rank test, *P* = 0.0001) (Fig. 4b). Persistence rates at 6 and 12 months post-index were approximately 74.0% (95% CI: 65.3, 80.3) and 54.5% (95% CI: 45.3, 62.8), respectively, in the latanoprost/timolol fixed-combination cohort and 46.2% (95% CI: 30.2, 60.7) and 25.6% (95% CI: 13.3, 39.9), respectively, in the counterpart cohort.

Additionally, when compared between classes of the fixed-combination therapies, that is, PG/BB vs. CAI/BB, at 12 months post-index, the persistence rate of the PG/BB group was approximately 8% higher than that of the CAI/ BB group (49.4% [95% CI: 42.9, 55.5] vs. 41.2% [95% CI: 29.5, 52.5]). However, it remained to be not statistically significant (log-rank test, *P* = 0.1454) (Fig. 4c).

## Discussion

The present study found that patients with fixed-combination therapy adhered to their therapies better than those with unfixed-combination therapy after switching from at least 12 months of monotherapy to a combination therapy with two drugs. Our findings are clinically important because no comparative study of adherence and persistence between fixed and unfixed-combination therapies has been reported in Japanese patients with glaucoma by using real-world data.

Most of the patients were prescribed with a combination of PG and BB in accordance with the guideline recommendation [9]. Overall, good adherence (PDC ≥ 80%) was noted in 58.8% of patients, lower than the results (72.4%) of a nationwide survey using a questionnaire in Japan [16]. In the fixed-combination cohort, 47.6% of patients continued the two-drug combination drugs at 12 months post-index, and 69.6% showed good adherence during the 12 months. Patients receiving a fixed-combination also had a higher 12-month persistence rate. The persistence rate of latanoprost/timolol fixed-combination was as high as 54.5%. Otherwise, most patients continued treatment using the same drops or the same class of drops, or switched to add-on therapy with the third drug (AA or CAI), suggesting that a fixed-combination was preferred. Although not significant, the PG/BB type had a slightly higher persistence rate than the CAI/BB type. Meanwhile, in the unfixed-combination cohort, only approximately 50% of the patients had good adherence. Furthermore, only 24.9% stayed on the unfixed two-drug combination therapy at 12 months post-index. Of note, the persistence rate of unfixed latanoprost plus timolol was as low as 25.6%. Therefore, if eye drops had the same combination, the fixed-combination therapy would achieve high adherence and persistence.

The major reason for the high adherence of the fixed combination therapy may be because they are more convenient for patients. An interval of 5 min or more is required when two ophthalmic solutions are used in unfixed combination eye drops, whereas two types of eye drops can easily be administered using a single eye drop in fixed combination drugs. Moreover, the number of eye drops and applications in fixed combinations are very small. Furthermore, to reduce the physical and economic burden on patients, physicians should explain to patients that the use of the fixed-combination therapies is advantageous because it requires less frequent medication and decreased exposure to preservatives (decreased risk of developing ocular surface diseases) [30, 31] with similar efficacies [15, 32–34], or possibly superior in daily practice [35], compared with unfixed-combination therapies. In an overseas clinical study, patients can better adhered with fixed-combination therapies than with unfixed-combination therapies [15]. Considering that good adherence to therapies is critical for controlling glaucoma progression [9, 36, 37], we believe that a fixed-combination therapy is an advantageous option for glaucoma treatment.

Given that the analyzed patients had received eye drop monotherapy for at least 1 year and continued to visit hospitals, they seemed to have been educated by doctors and have possibly understood the importance of continuing treatment with eye drops. However, many patients were nonadherent to the index therapy. In fact, an unfixed-combination therapy was consistently a risk factor for adherence. Furthermore, a high HbA1c level, but not diabetes mellitus, was another risk factor for nonadherence to the treatment. Considering the small sample size and numerous missing data, HbA1c is carefully concluded as a risk factor. However, diabetic patients with higher HbA1c levels are apt to be nonadherent to the therapy because a higher HbA1c level; i.e., poor glycemic control can be partly explained by poor adherence to antidiabetic medications and medical visits [38–41]. Type 2 diabetes mellitus, as well as glaucoma, is a chronic disease in which patients may have few symptoms despite having complex medication. If patients have complications, they need to take more pills at different times as per medicine. These patients may pay minimal attention to eye drops, leading to glaucoma progression. Therefore, glaucoma should be treated with continuous intervention in patients with diabetes mellitus, especially patients with poor glycemic control.

Moreover, other common risk factors were previously identified [12–14]. All of these risk factors need to be solved by education, communication with patients, and patient training in improving proficiency of self-administration of eye drops [42].

Specific reasons on why physicians prescribed unfixed combinations for certain patients would be interesting to identify. Such reasons may include a risk of adverse events caused by the concentration of the fixed-combination, preservative formulations, and dosage adjustment of eye drops. If so, developing a new fixed-combination therapy would be worthwhile.

This study, however, has limitations. First, this study assumes that patients took eye drops for the number of days that they were prescribed, because the claims database does not track whether the patient actually administrated the eye drops as prescribed. However, we set a 30-day grace period so that the PDC did not underestimate adherence. Second, the study did not consider baseline PDC in patients that had being continuing monotherapy for 12 months prior to the index date, although baseline may or may not differ between the groups. Third, we used the MinaCare database to gather healthcare information covering nearly 2 million individuals, of which 90% were aged 20–59 years who worked for a nationwide large company, of which, most individuals lived in Tokyo or large cities in East Japan, and their dependents in private corporate health insurance societies [24, 25]. Therefore, regional and income biases might have affected the results. Moreover, only few elderly patients were included in this study. However, this study is worth considering for treating glaucoma because young patients are more likely to have glaucoma progression during their long life-spans. Fourth, the patients between the two cohorts possessed an uncontrolled nature, which includes the numerous drug types used as monotherapy during the pre-index period and the severity of glaucoma. Although background information and baseline characteristics were limited, they were similar between the two cohorts. Finally, the present analyses were built according to the assumption that all of the claimed drugs were used by the patients. To address these limitations, we need to conduct further studies using the real-world data combined with clinical data.

## Conclusions

The present results using the real-world data showed that city patients who received fixed-combination therapies exhibited better adherence to medications compared with those who received unfixed-combination therapies. A high HbA1c level was one risk factor for nonadherence. Therefore, a fixed-combination therapy would better serve as a glaucoma therapy, considering that adherence to the therapy is a key for effectively preventing glaucoma progression.

## Data Availability

The data that support the findings of this study are available from MinaCare but restrictions apply to the availability of these data, which were used under license for the current study, Therefore, the data are not publicly available. The data are however available from the authors upon reasonable request and with the permission of MinaCare.

## Abbreviations

AA: α2-agonist
BB: β-blocker
CAI: Carbonic anhydrase inhibitor
CI: Confidence interval
HbA1c: Hemoglobin A1c
IOP: Intraocular pressure
MIGS: Minimally invasive glaucoma surgery
NS: Not significant
OR: Odds ratio
PDC: Proportion of days covered
PG: Prostaglandin analog
SD: Standard deviation
SLT: Selective laser trabeculoplasty
